# Perioperative Cumulative Fluid Balance and Its Association with an Increase in Costs after Major Surgery

**DOI:** 10.3390/jcm7090227

**Published:** 2018-08-21

**Authors:** Tak Kyu Oh, Jung-Won Hwang, Young-Tae Jeon, Sang-Hwan Do

**Affiliations:** 1 Department of Anesthesiology and Pain Medicine, Seoul National University Bundang Hospital, Gumi-ro 173 Beon-gil, Bundang-gu, Seongnam 13620, Korea; jungwon@snubh.org (J.-W.H.); ytjeon@snubh.org (Y.-T.J.); shdo@snu.ac.kr (S.-H.D.); 2 Department of Anesthesiology and Pain Medicine, College of Medicine, Seoul National University, 103 Daehak-ro, Jongno-gu, Seoul 03080, Korea

**Keywords:** costs and cost analysis, general surgery, hospitals

## Abstract

Positive fluid balance (FB) during the perioperative period may increase the incidence of postoperative complications, which may lead to longer hospitalization and higher hospital costs. However, a definitive association between positive FB and hospital costs has not yet been established. This retrospective observational study examined the association between perioperative FB and hospital costs of patients who underwent major surgical procedures. Medical records of patients who underwent major surgery (surgery time >2 h, estimated blood loss >500 mL) from January 2010 to December 2017 were analyzed to determine the associations between calculated FB (%, total input fluid—output fluid in liter/weight (kg) at admission) and total hospital cost ($). The analysis included medical data of 7010 patients. Multivariable linear regression analyses showed that a 1% increase in FB in postoperative day (POD) 0 (24 h), 0–1 (48 h), 0–2 (72 h), and 0–3 (96 h) significantly increased the total cost by $967.8 (95% confidence interval [CI]: 803.4–1132.1), $688.8 (95% CI: 566.3–811.2), $591 (95% CI: 485.7–696.4), and $434.2 (95% CI: 349.4–519.1), respectively (all *p* < 0.001). Perioperative cumulative FB was positively associated with hospital costs of patients who underwent major surgery.

## 1. Introduction

Perioperative fluid therapy is an important factor that may affect the prognosis of patients undergoing surgery [1,2,3] and, therefore, it is an indispensable issue in clinical practice [4]. Several perioperative fluid management strategies have been studied so far; liberal and restrictive fluid therapies are two widely used regimens [5]. Restrictive fluid therapy has been demonstrated to prevent positive fluid balance (FB) and achieve a “zero FB” status, and to reduce the incidence of postoperative complications [6,7] as well as the length of hospitalization [5,8]. As such, reductions are closely related to lowering total hospital costs; hence, perioperative fluid management is also an important economic issue [9,10]. Thus, perioperative fluid strategy to prevent positive FB is an important factor that is associated with postoperative complications, length of hospitalization, and hospital costs of a surgical population.

In recent years, studies have begun to use weight-based FB (%), which considers not only fluid input value, but also the amount of fluid output and patients’ weight at admission [11,12]. Such FB calculations in percentages using patients’ weight have been effectively used in the fluid management of critically ill or pediatric patients, who are more sensitive to fluid imbalance. Using weight-based FB allows for a more effective prediction of the associations between positive FB and postoperative outcomes of a surgical population, as well as hospital costs. However, a definitive association between FB and hospital costs of a surgical population has not yet been established. Thus, the present study aimed to examine the association between weight-based FB and hospital costs of patients who underwent major surgical procedures. We hypothesized that positive FB would be positively associated with total hospital costs and daily costs.

## 2. Materials and Methods

This study was approved by the Institutional Review Board (IRB) of Seoul National University Bundang Hospital (SNUBH) (approval number: B-1803/459-105, approval date: 12 March 2018). The requirement for informed consent was waived by the IRB as this was a retrospective cohort analysis.

### 2.1. Patients

Medical records of patients aged 19 years or older who underwent a major surgery at SNUBH from January 2010 to December 2017 were retrospectively analyzed. Major surgery was defined as a surgery that exceeded two hours with an estimated blood loss (EBL) of more than 500 mL. Incomplete or missing medical records were excluded from the analysis.

### 2.2. Calculation of FB (%) in Postoperative Day 0–3

Each patient’s cumulative FB was expressed as percentage based on the following four times: (1) postoperative day (POD) 0 (24 h), (2) POD 0–1 (48 h), (3) POD 0–2 (72 h), and (4) POD 0–3 (96 h). We used the following equation from previous studies to calculate the cumulative FB (%) [11,12]:

Cumulative FB (%) = (Cumulative Fluid Input − Output) in liters × 100/Hospital Admission Weight (kg)

Fluid input included all fluids infused by intravenous or enteral routes for maintenance and resuscitation: colloids, crystalloids, blood products, drug infusions, and enteral and parenteral nutrition. Fluid output included all body fluids.

### 2.3. Calculation of Costs for Major Surgical Procedures

Most patients in Korea are enrolled in the National Health Insurance Service (NHIS) provided by the Korean government as required by law, which covers approximately two-thirds of essential medical costs within hospitals [13]. In the present study, each patient’s total hospital cost was calculated by summing up the patient’s out-of-pocket cost and costs covered by the NHIS. Daily cost was defined as the value obtained by dividing the patient’s total hospital cost by the length of hospitalization. In addition, non-coverage by the NHIS (%) was defined as the value from the following equation: patient’s out-of-pocket cost/the total hospital cost × 100. All costs were first computed in Korean currency, won (₩), and then converted to the equivalent in U.S. dollars ($) based on the exchange rate of 1060 won = 1 dollar to express the unit of costs in dollars.

### 2.4. Measurement and Outcome

Demographic information (age, body mass index, and sex), preoperative comorbidities, and surgery-related information of the patients were extracted. The collected preoperative comorbidity information included hypertension, diabetes mellitus, history of ischemic heart disease and cerebrovascular disease, liver disease (hepatitis, liver cirrhosis, hepatocellular carcinoma), and cancer. Types of surgery were divided into the following five groups: (1) general surgery, (2) cardiovascular surgery, (3) thoracic surgery, (4) obstetric, gynecologic, or urologic surgery, and (5) plastic/orthopedic surgery or neurosurgery. In addition, liver and kidney transplantations were included in the general surgery group, lung and heart-lung transplantations were included in the thoracic surgery group, and heart transplantation was included in the cardiovascular surgery group. All medical records were extracted by a medical record technician who was blinded to the purpose of the study.

The primary outcome of this study was to examine the association between perioperative FB and total and daily hospital costs. We also analyzed the effect of patients’ out-of-pocket medical costs on overall costs.

### 2.5. Statistical Analysis

Continuous and categorical variables of the patients’ baseline characteristics were expressed as mean with standard deviation (SD) and number with percentage, respectively. First, a restricted cubic spline was used to characterize the pattern of change in total hospital cost ($) and daily cost ($) according to FB (%) increase. Next, we conducted univariable linear regression analyses to examine the independent association of each variable with total costs and daily costs. From univariable linear regression models, variables with *p* < 0.1 were included as covariates in multivariable linear regression analyses. The potential presence of multicollinearity among each FB (%) (POD 0, POD 0–1, POD 0–2, and POD 0–3) was considered, and four multivariable linear regression models were developed for each FB. Using the above method, we performed a multivariable linear regression analysis according to the five surgery types for an additional subgroup analysis. All data were analyzed using IBM SPSS version 24.0 (IBM Corp., Armonk, NY, USA), and statistical significance was assumed at *p* < 0.05.

## 3. Results

**Figure 1:**
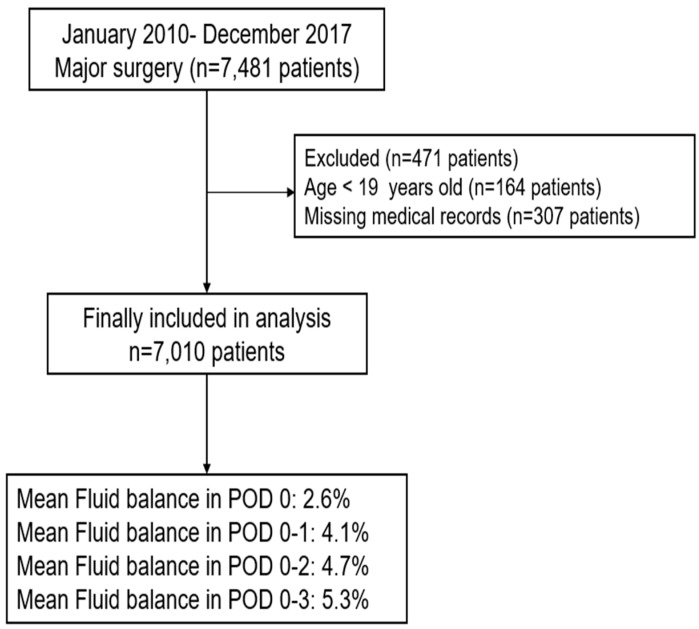
Flowchart for patient selection. POD: postoperative day.

In total, 7481 patients underwent major surgery that exceeded two hours and with an EBL of more than 500 mL from January 2010 to December 2017. Of these, 164 patients under 19 years of age and 307 patients with incomplete or missing medical records were excluded from the analysis. Medical records of the final sample of 7010 patients were analyzed. Patients’ FB (%) in POD 0, 0–1, 0–2, and 0–3 were 2.6 (3.1), 4.1 (3.1), 4.7 (5.0), and 5.3 (6.1), respectively (Figure 1). Baseline characteristics of the patients are shown in Table 1. The total and daily costs incurred by patients who underwent major surgery were $19,156.9 ($22,633.9) and $1198.6 (904.1), respectively. Of the total cost, non-coverage by the NHIS was 35.3% (15.9%).

### 3.1. Total and Daily Costs According to FB (%) in POD 0–3

**Figure 2:**
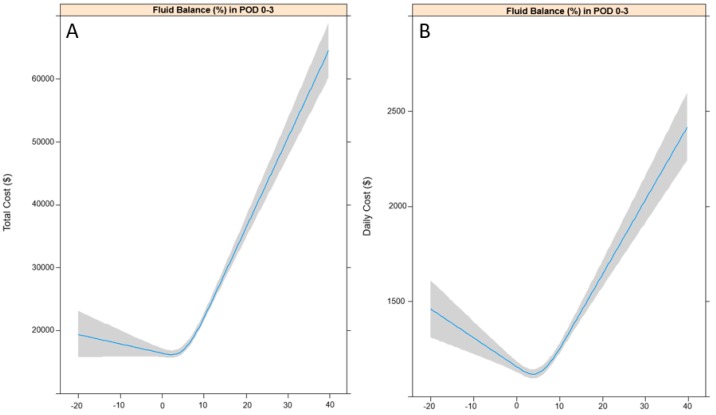
Restricted cubic splines for total costs (**A**) and daily costs (**B**) according to fluid balance in POD 0–3. POD: postoperative day.

The patterns of change in total and daily costs according to FB (%) in POD 0–3 are shown in Figure 2A,B. Total and daily costs increased as FB in POD 0–3 increased. The results of univariable linear regression analyses are shown in Table 2, while the results of the multivariable linear regression analysis, after controlling for variables selected from the univariable linear regression model, are shown in Table 3. A 1% increase in FB in POD 0 (24 h), 0–1 (48 h), 0–2 (72 h), and 0–3 (96 h) all significantly increased total costs by $967.8 (95% CI: 803.4–1132.1), $688.8 (95% CI: 566.3–811.2), $591 (95% CI: 485.7–696.4), and $434.2 (95% CI: 349.4–519.1), respectively (all *p* < 0.001). Similarly, a 1% increase in FB in POD 0 (24 h), 0–1 (48 h), 0–2 (72 h), and 0–3 (96 h) all significantly increased daily costs by $19.9 (95% CI: 13.5–26.2), $21.3 (95% CI: 14.5–27.0), $18.9 (95% CI: 15.0–22.9), and $14.6 (95% CI: 11.4–17.8), respectively (all *p* < 0.001). In addition, a 1% increase in non-coverage by the NHIS was associated with a $66.3 reduction in total cost (95% CI: −97.8, −34.7) and a $5.8 reduction in daily cost (95% CI: −7.0, −4.6) (*p* < 0.001).

### 3.2. Subgroup Analysis According to Type of Surgery

The results of multivariable linear regression analyses of total cost ($) according to FB in POD 0–3 by each type of surgery are shown in Table 4. As FB increased, the total cost increased for all surgery types (coefficient > 0, *p* < 0.001), and this association was the highest in the thoracic surgery group (coefficient > 2000), followed by the cardiovascular surgery group (coefficient > 600, *p* < 0.001).

## 4. Discussion

Our study demonstrated that there is a significant positive association between perioperative positive FB and total and daily costs. Increases in costs resulted in relatively higher FB (%) in POD 0 (24 h) and POD 0–1 (48 h) than in POD 0–2 (72 h) and POD 0–3 (96 h). In addition, the strongest association was observed in patients who underwent relatively more expensive and invasive cardiothoracic surgery. As improving surgical quality and reducing hospital costs are two important challenges facing hospitals and the government [14], our study’s findings are important because we revealed the association between perioperative positive FB and hospital costs of a surgical population. Moreover, it is worth noting that we used weight-based FB that considers patients’ weight at the time of admission in our analysis to show its association with hospital costs.

Furthermore, the results of this study may be important from the perspective of enhanced recovery after surgery (ERAS) [15]. There is controversy regarding the optimal fluid management strategy in the perioperative period, and the target of achieving zero FB in the perioperative period is usually recommended for ERAS [16]. In addition, the implementation of ERAS protocol can reduce hospital costs after pancreaticoduodenectomy [17] and colorectal surgery [18]. Regarding this point, our finding that positive FB is associated with an increase in hospital costs after major surgery is consistent with the findings of previous studies [16,17,18]. However, we did not consider goal-directed fluid therapy (GDFT), because our institution did not use any fluid management protocol regarding GDFT during the study period. Considering that GDFT is known to reduce length of hospitalization after major surgery [19], it can also help reduce hospital costs. Therefore, it is necessary to conduct further research on the relationship between GDFT and surgery costs.

We hypothesized that positive FB may be associated with increased costs of a surgical population based on findings from previous studies. First, perioperative FB has been reported to be associated with increased postoperative complications [6], which results in higher hospital costs [9,10]. Thus, we hypothesized that perioperative FB would be positively associated with hospital costs, which was effectively demonstrated in our study. Second, surgical bleeding, which is one of the major causes of fluid imbalance during surgery, may also affect hospital costs. Indeed, it has been reported that the length and costs of hospitalization increase as the severity of surgical bleeding increases during left-ventricular assist device surgery [20]. In summary, our study showed that FB may be a useful clinical index when implementing a clinical pathway to reduce hospital costs [21].

Our study is noteworthy in that it was conducted in Korea where the NHIS covers an average of 65% of all medical costs. In our study, the SD of the NHIS coverage was relatively small (15%), and since all patients had similar insurance coverage, our findings reflect the effect of positive FB, rather than that of individual insurance statuses, on medical costs. If our study had analyzed a population of patients with different insurance statuses, the results would have been greatly affected by the differences in each patient’s insurance status. In fact, a cohort study in the United States reported that patients with relatively less insurance coverage are at greater risk of developing postoperative complications after colorectal cancer surgery [22], revealing insurance status to be an important factor that affects both the rate of complications and hospital costs. Thus, the fact that our study used medical records of patients with a similar insurance coverage under the NHIS adds significance to the association found between positive FB and hospital costs.

Another interesting finding of the present study is that as the percentage of non-coverage by the NHIS increased or, in other words, as the patients’ out-of-pocket costs increased, the total costs independently decreased. As this result was obtained after controlling for the variables, including type of surgery, surgery time, age, and comorbidities, the “moral hazard” effect of health insurance may have influenced the results [23]. Moral hazards describe the excessive use of and expenditure on medical service by health consumers, or patients, when they have insurance coverage, and it is regarded as an important economic issue [24]. In the present study, there was a decreasing pattern in total and daily costs when the patients’ out-of-pocket costs increased, which may be partly attributed to the moral hazard effect of health insurance. Further research is needed to explore this pattern.

Our study has several limitations. First, selection bias may have been present as a limitation of a retrospective observational study design. We tried to circumvent this limitation, however, by having a medical record technician, who was blinded to the purpose of the study, extract all medical records used for the study. Second, since it was a single-center study, the generalizability may be limited. Lastly, we did not consider fluid evaporation in FB calculation, given the heterogeneity of the surgical procedures. Despite these limitations, our study was the first to show that an increase in FB during the perioperative period is independently associated with hospital costs.

## 5. Conclusions

Our study showed that perioperative FB is positively associated with hospital costs of patients who underwent major surgery. Our study’s findings are important because we revealed the association between perioperative positive FB and hospital costs of a surgical population. Future studies on the relationship between GDFT for surgery and hospital costs are necessary.

## Figures and Tables

**Table 1:** Characteristics of patients who received major surgical procedures in 2010–2017.

Variables	Total (*n* = 7010)	Mean (SD)
Age, year		59.2 (14.5)
Body mass index, kg m^−2^		24.2 (3.7)
Sex: male	3657 (52.2%)	
Preoperative comorbidities		
Hypertension	2493 (35.6%)	
Diabete mellitus	722 (10.2%)	
Ischemic heart disease	168 (2.5%)	
Cerebrovascular disease	187 (2.7%)	
Chronic kidney disease	125 (1.8%)	
Liver disease (Hepatitis, LC, HCC)	448 (6.4%)	
Cancer	482 (6.9%)	
Information regarding surgical procedures		
Surgery time, min		301.7 (137.9)
Estimated blood loss, mL		1172.2 (1683.7)
Length of hospital stay, da		19.5 (25.1)
Type of surgery		
General surgery	2190 (31.3%)	
Cardiovascular surgery	764 (10.9%)	
Thoracic surgery	119 (1.7%)	
Obstetrics, Gynecologic, Urologic surgery	1564 (22.3%)	
Plastic, Orthopedic, Neurosurgery	2373 (33.8%)	
Years at surgery		
2010–2013	1222 (17.4%)	
2013–2015	1980 (28.2%)	
2015–2017	3808 (54.3%)	
Fluid balance (%) in postoperative day 0–3		
Postoperative day 0 (24 h)		2.6 (3.1)
Postoperative day 0–1 (48 h)		4.1 (4.3)
Postoperative day 0–2 (72 h)		4.7 (5.0)
Postoperative day 0–3 (96 h)		5.3 (6.1)
Total cost ($)		19,156.9 (22,633.9)
Daily cost ($)		1198.6 (904.1)
Non-coverage: National Health Insurance Service (%)		35.3 (15.9)

Fluid balance (%): (Total input fluid—total output fluid) in liter × weight in admission (kg)^−1^ × 100 SD, standard deviation; LC, liver cirrhosis; HCC, hepatocellular carcinoma.

**Table 2:** Univariable linear regression analysis for total and daily cost ($) in patients who received major surgical procedures.

Characteristics	Total Cost ($)		Daily Cost ($)	
	Coefficient (95% CI)	*p*	Coefficient (95% CI)	*p*
Sex: male (vs. female)	3889.9 (2832.5, 4947.2)	<0.001	85.4 (43.0, 127.8)	<0.001
Age, year	112.0 (75.6, 148.4)	<0.001	1.2 (−0.3, 2.6)	0.119
Body mass index, kg m^−2^	−321.3 (−463.4, −179.3)	<0.001	0.1 (−5.6, 5.8)	0.965
Hypertension	2129.9 (1024.0, 3235.8)	<0.001	66.7 (22.4, 110.9)	0.003
Diabete mellitus	9073.8 (6685.3, 11462.4)	<0.001	197.9 (102.2, 293.6)	<0.001
Cerebrovascular disease	5343.8 (2509.5, 8178.0)	<0.001	123.9 (−7.8, 255.6)	0.065
Ischemic heart disease	10797.3 (7341.4, 14,253.3)	<0.001	629.1 (491.0, 767.1)	<0.001
Chronic kidney disease	14244.9 (10,254.2, 18,235.7)	<0.001	312.8 (152.3, 473.2)	<0.001
Liver disease	13831.4 (11,688.9, 15,973.8)	<0.001	397.5 (311.4, 483.5)	<0.001
Cancer	732.6 (−1361.7, 2827.0)	0.493	−33.5 (−117.2, 50.2)	0.432
Surgery time, min	34.1 (30.3, 37.8)	<0.001	0.5 (0.3, 0.7)	<0.001
Estimated blood loss, mL	3.6 (3.3, 3.9)	<0.001	0.1 (0.1, 0.1)	<0.001
Type of surgery				
General surgery	1		1	
Cardiovascular surgery	14620.4 (12,897.1, 16,343.8)	<0.001	1072.6 (1005.1,1140.1	<0.001
Thoracic surgery	−13734.4 (−15,092.7, −12,376.1)	<0.001	199.2 (48.0, 350.3)	0.010
OG, Urologic surgery	−6502.8 (−7718.1, −5287.5)	<0.001	−304.5 (−357.7, −251.3)	<0.001
PS, OS, NS	8411.7 (4551.1, 12,272.3)	<0.001	−2.6 (−50.2, 45.0)	0.914
Surgery in 2010–2012	1		1	
Surgery in 2013–2015	4360.7 (2754.4, 5967.1)	<0.001	250.8 (188.2, 313.4)	<0.001
Surgery in 2016–2017	6218.5 (4766.8, 7670.2)	<0.001	556.7 (500.1, 613.3)	<0.001
FB (%) in POD 0 (24 h)	1665.9 (1501.6, 1830.3)	<0.001	34.0 (27.3, 40.7)	<0.001
FB (%) in POD 0–1 (48 h)	1224.7 (1104.8, 1344.5)	<0.001	30.0 (25.1, 34.8)	<0.001
FB (%) in POD 0–2 (72 h)	999.1 (895.4, 1102.8)	<0.001	22.4 (18.2, 26.6)	<0.001
FB (%) in POD 0–3 (96 h)	728.0 (642.7, 813.3)	<0.001	15.1 (11.6, 18.5)	<0.001
Non–coverage: NHIS (%)	−285.7 (−318.5, −253.0)	<0.001	−5.1 (−6.4, −3.8)	<0.001

Variables with *p* < 0.1 were included multivariate linear regression analysis in Table 3. OG, obstetrics and gynecologic; PS, plastic surgery; OS, orthopedic surgery; NS, neurosurgery; FB, fluid balance; POD, postoperative day; NHIS, National Health Insurance Service.

**Table 3:** Multivariable linear regression analysis for total and daily cost ($) in patients who received major surgical procedures.

Variables	Multivariable Model (Total Cost, $)		Multivariable Model (Daily Cost, $)	
	Coefficient (95% CI)	*p-*Value	Coefficient (95% CI)	*p-*Value
FB (%) in POD 0 (24 h) ^a^	967.8 (803.4, 1132.1)	<0.001	19.9 (13.5, 26.2)	<0.001
FB (%) in POD 0–1 (48 h) ^b^	688.8 (566.3, 811.2)	<0.001	21.3 (4.5, 7.0)	<0.001
FB (%) in POD 0–2 (72 h) ^c^	591.1 (485.7, 696.4)	<0.001	18.9 (15.0, 22.9)	<0.001
FB (%) in POD 0–3 (96 h) ^d^	434.2 (349.4, 519.1)	<0.001	14.6 (11.4, 17.8)	<0.001
Non-coverage: NHIS (%) ^d^	−66.3 (−97.8, −34.7)	<0.001	−5.8 (−4.6, −7.0)	<0.001

All covariates of *p* < 0.1 in each univariable linear regression models (Table 2) were included in four multivariable linear regression analysis (a–d). FB (%) in POD 0, 0–1, 0–2. And 0–3 were included in each different multivariable linear regression models (a–d) to avoid interactions between variables regarding FB (%). FB, fluid balance; NHIS, National Health Insurance Service.

**Table 4:** Multivariable linear regression analysis for total cost ($) in patients according to major surgical procedures.

Variables	Total Costs ($)	*p*-Value
	Coefficient (95% CI)	
General surgery		
Fluid balance (%) POD 0 (24 h)	1214.8 (909.9, 1519.6)	<0.001
Fluid balance (%) POD 0–1 (48 h)	881.5 (651.5, 1111.5)	<0.001
Fluid balance (%) POD 0–2 (72 h)	828.0 (629.0, 1027.0)	<0.001
Fluid balance (%) POD 0–3 (96 h)	653.9 (492.3, 815.5)	<0.001
Cardiovascular surgery		
Fluid balance (%) POD 0 (24 h)	2088.3 (1409.3, 2767.3)	<0.001
Fluid balance (%) POD 0–1 (48 h)	1524.6 (954.7, 2094.5)	<0.001
Fluid balance (%) POD 0–2 (72 h)	1099.4 (587.3, 1611.6)	<0.001
Fluid balance (%) POD 0–3 (96 h)	634.4 (220.7, 1048.1)	0.003
Thoracic surgery		
Fluid balance (%) POD 0 (24 h)	9685.0 (5810.9, 13,559.1)	<0.001
Fluid balance (%) POD 0–1 (48 h)	6194.5 (3407.9, 8981.0)	<0.001
Fluid balance (%) POD 0–2 (72 h)	3982.7 (1733.6, 6231.7)	0.001
Fluid balance (%) POD 0–3 (96 h)	2272.5 (571.3, 3973.6)	0.009
Obstetrics, Gynecologic, Urologic surgery		
Fluid balance (%) POD 0 (24 h)	210.9 (106.8, 314.9)	<0.001
Fluid balance (%) POD 0–1 (48 h)	140.8 (71.4, 210.3)	<0.001
Fluid balance (%) POD 0–2 (72 h)	112.9 (54.9, 170.9)	<0.001
Fluid balance (%) POD 0–3 (96 h)	82.3 (35.7, 128.8)	0.001
Plastic, Orthopedic, Neurosurgery		
Fluid balance (%) POD 0 (24 h)	314.6 (1.342, 627.9)	0.049
Fluid balance (%) POD 0–1 (48 h)	262.9 (11.5, 514.3)	0.040
Fluid balance (%) POD 0–2 (72 h)	312.1 (97.7, 526.6)	0.004
Fluid balance (%) POD 0–3 (96 h)	255.1 (90.5, 419.7)	0.002

All Covariates of *p* < 0.1 in univariable linear regression analysis for total cost ($) were included in multivariable linear regression analysis. POD, postoperative day.
